# Self-Catalyzed CdTe Wires

**DOI:** 10.3390/nano8050274

**Published:** 2018-04-25

**Authors:** Tom Baines, Giorgos Papageorgiou, Oliver S. Hutter, Leon Bowen, Ken Durose, Jonathan D. Major

**Affiliations:** 1 Stephenson Institute for Renewable Energy, Physics Department, University of Liverpool, Liverpool L69 7XF, UK; g.papageorgiou@swansea.ac.uk (G.P.); O.S.Hutter@liverpool.ac.uk (O.S.H.); dph0kd@liverpool.ac.uk (K.D.); jonmajor@liverpool.ac.uk (J.D.M.); 2 Department of Physics, G.J. Russell Microscopy Facility, Durham University, South Road, Durham DH1 3LE, UK; leon.bowen@durham.ac.uk

**Keywords:** CdTe, self-catalysed, wires

## Abstract

CdTe wires have been fabricated via a catalyst free method using the industrially scalable physical vapor deposition technique close space sublimation. Wire growth was shown to be highly dependent on surface roughness and deposition pressure, with only low roughness surfaces being capable of producing wires. Growth of wires is highly (111) oriented and is inferred to occur via a vapor-solid-solid growth mechanism, wherein a CdTe seed particle acts to template the growth. Such seed particles are visible as wire caps and have been characterized via energy dispersive X-ray analysis to establish they are single phase CdTe, hence validating the self-catalysation route. Cathodoluminescence analysis demonstrates that CdTe wires exhibited a much lower level of recombination when compared to a planar CdTe film, which is highly beneficial for semiconductor applications.

## 1. Introduction

Due to their unique properties, semiconductor nano and microwires have attracted a lot of interest for optoelectronic devices. Direct band gap wires offer enhanced performance due to an increased effective surface area and reduced recombination [1,2,3]. CdTe is a direct semiconductor widely utilized for photovoltaics (PV) as a thin film absorber, owing to its near optimal band gap for PV of 1.5 eV and ease of deposition owing to its comparatively simple phase chemistry [4]. CdTe can be produced by a variety of techniques, such as physical vapor deposition (PVD) [5], chemical vapor deposition (CVD) [6], molecular beam epitaxy (MBE) [7] and solution phase synthesis [8]. It has been suggested that solar cells based on CdTe wires may exhibit a higher performance, due to the advantageous carrier transport properties of the wires. However, the fabrication of CdTe wires is far more challenging than for the planar films. CdTe wires grown by PVD or CVD techniques typically occur via a vapor-liquid-solid (VLS) mechanism necessitating a metal catalyst seed particle to facilitate wire growth [9]. The use of catalysts is problematic when attempting to create device quality material. Au and Bi have been used to successfully synthesize CdTe wires [9,10], however both have been established as deep level recombination centers in CdTe, compromising performance [11,12]. Catalyst-free CdTe wires are therefore preferable to minimize the defect content. Prior work on catalyst free CdTe wires primarily consists of two approaches (i) a solution based method grown via a solution-liquid-solid (SLS) mechanism [13], or (ii) a template assisted electrodeposition route using, for example, an aluminum oxide film as the template [14]. Both of these techniques have their disadvantages; they often require the use of solvents like oleylamine or complex patterning steps for the template layers. Wires produced by solution based methods are also often difficult to incorporate into device structures as many applications require vertically aligned wires projecting from the surface of the substrate [15].

The only prior report of self-catalyzed CdTe wires via PVD was from Wang et al. who reported growth via a thermal chemical method on an ITO substrate via a proposed vapor-solid-solid (VSS) mechanism [16], where the substrate was placed in a alumina vacuum tube furnace and CdTe was deposited at 2 × 10^−1^ mbar and 700 °C. Nanorods were observed to grow from a single CdTe seed particle formed on the substrate surface. The (111) zincblende crystal surface is more active than the substrate leading to continuous stacking along the CdTe (111) plane resulting in a preferred 1D growth [16]. It is worth noting that we use the term catalysis in this context to infer the CdTe seed particle is the cause for the wire formation, rather than the reaction rate is being increased.

In this work we present self-catalyzed wire growth via a proven scalable deposition route, close space sublimation (CSS) [5] on Mo substrates and as such this work has direct relevance to potential device production [17]. The influence of growth pressure and substrate surface roughness on wire growth were investigated with characterization by scanning electron microscopy (SEM), X-ray diffraction (XRD), energy dispersive X-ray (EDX) spectroscopy and cathodoluminescence (CL).

## 2. Materials and Methods

A range of substrates were used in this work; uncoated soda-lime glass (SLG), fluorine doped tin oxide (FTO) coated SLG “TEC 6” glass (NSG Ltd., St. Helens, UK) and 0.1 mm Mo foil substrates (Advent, 99.95% pure, Oxford, UK). All substrates were washed with isopropyl alcohol (IPA) and de-ionized (DI) water, then ultrasonically cleaned in DI water prior to deposition. 250 nm (0.5 Ω/sq) Mo films were grown onto the glass substrates via Direct Current (DC) magnetron sputtering at 400 °C using an Ar plasma. 6–8 μm CdTe (Alfa Aesar, 99.99% pure, Lancashire, UK) was deposited via CSS in an N_2_ ambient at a variety of pressures and at source and substrate temperatures of 650 °C and 550 °C respectively.

Atomic force microscopy (AFM) was carried out using a Veeco Innova Bruker atomic force microscope (Bruker, CA, USA) in contact mode. XRD measurements were performed using a PANalytical X’pert PRO X-ray diffractometer (PANalytical B. V., Eindhoven, The Netherlands) at room temperature, using CuKα1 line as the X-ray source. SEM images were taken using a JSM-7001F microscope from JEOL with EDX spectrometer (JEOL, Tokyo, Japan). CL spectra was measured with a Hitachi SU-70 SEM (Hitachi, Tokyo, Japan) operating at 12 keV together with a Gatan MonoCL system (Gatan, CA, USA) for CL detection. The pixel dwell time for the panchromatic was 4 s.

## 3. Results and Discussion

**Figure 1:**
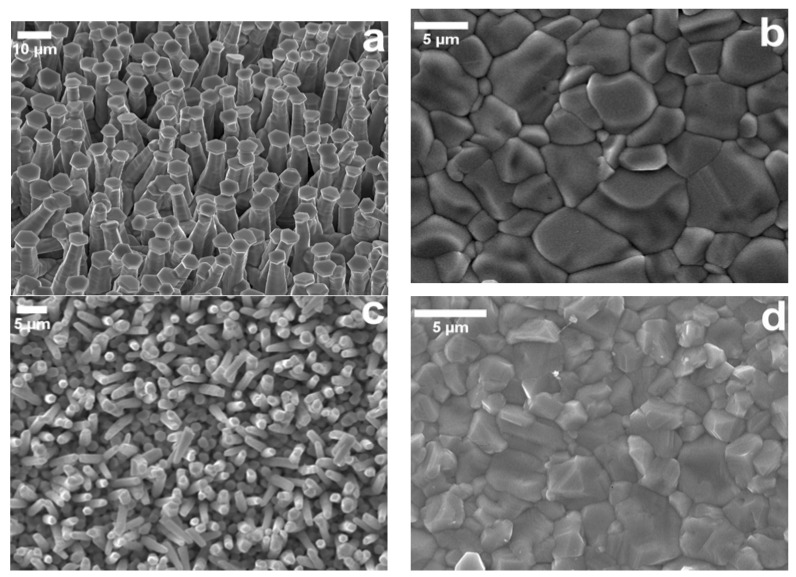
SEM images showing the effect of the substrate on wire growth. CdTe deposition was performed under identical growth conditions on: (**a**) SLG/Mo; (**b**) Mo Foil; (**c**) SLG; and (**d**) SLG/FTO/Mo.

The preferable implementation for CdTe wires in a solar cell structure is via the “substrate” cell structure [17], wherein the CdTe component must be deposited on top of a suitable back contact medium. Typically for substrate CdTe devices the back contact material is Mo as its thermal expansion coefficient is close to that of CdTe and its use is well established for other thin film technologies such as Copper Indium Gallium Selenide (CIGS) and Copper Zinc Tin Sulfide (CZTS) [18,19], although there are some issues with regards to generation of an Ohmic contact [17]. We therefore focused on producing wires on a Mo surface as this is the first step towards wire cell development. We considered two substrate options: Mo coated SLG, the typical route for CIGS/CZTS devices, or growth directly onto Mo foil, which is of interest due to the potential to produce flexible solar cells [18]. Figure 1a,b shows SEM images of CdTe growth on SLG/Mo and Mo foil respectively, using deposition conditions equivalent to that for our standard CdS/CdTe superstrate cell platform [20]. For growth on Mo foil (Figure 1b) we observe what we would classify as a “typical” CdTe thin film growth for these conditions, i.e., complete coverage of the substrate with a grain size 1–5 μm [21]. For growth on the SLG/Mo substrates under identical growth conditions we observed the formation of a field of self-catalysed wires. The wires show an ordered lateral growth with an average diameter of 3.5 ± 0.3 μm, average length of 15.9 ± 3.8 μm and clear evidence of a hexagonal cap to the wires. Prior nucleation and growth studies have identified similar hexagonal CdTe islands to form during the early stages of CSS deposition [22].

From this and the microscopy images we infer a vapor-solid-solid (VSS) growth mode, wherein hexagonal seed crystals are initially formed on the surface then subsequently act as a template for wire growth, following a similar mechanism to the one proposed by Wang et al. [16,23]. Such mechanisms are likely to be highly sensitive to the mobility of adatoms on the surface, which will influence the critical nucleus size [24] and thus the formation of any surface-stable seed crystal, as well as the rate of material flux to feed wire growth. The lack of wire formation for growth on the Mo foil can therefore be attributed to one of two factors either (i) the surface energy of the foil is significantly different compared to SLG/Mo, thus influencing the adatom lifetime on the surface and altering the growth mode [25] or (ii) as the Mo foil is significantly rougher than Mo/SLG (71.98 nm Root Mean Square (RMS) vs. 8.0 nm RMS roughness, Figure S1 in supporting information shows the three dimensional AFM images of the substrates surface) the increased roughness is hampering adatom diffusion disrupting the formation of seed particles.

A route to better understand the limiting factor was to utilize identical growth conditions but using uncoated SLG and SLG/FTO/Mo coated substrates. These substrates were selected in particular to separate the influence of the deposition surface from the surface roughness. For deposition on SLG the surface bonding energy is completely different compared to SLG/Mo but the roughness is even lower. FTO coated glass is stable at high temperatures due to its CVD deposition route and has higher roughness than SLG, 16.17 nm RMS (Figure S1), but by coating it with sputtered Mo is offers an identical surface to SLG/Mo only with increased roughness. Via comparison of growth on these substrates it allows us to separate the influence of the Mo layer from the roughness. SEM images for growth on SLG and SLG/FTO/Mo are shown in Figure 1c,d respectively. We observe the formation of wires on the SLG surface but thin-film style growth on the SLG/FTO/Mo surface, hence it is apparent that surface roughness is the controlling factor for wire formation. For the highly smooth Mo-free SLG surface, wires have formed with smaller dimensions than that observed for the SLG/Mo surface. This again demonstrates the influence of roughness but additionally allows us to rule out the Mo film acting as a catalyst layer and clearly demonstrates the wires are self-catalysed. For the rougher SLG/FTO/Mo surface, growth has reverted to thin films, albeit of slightly smaller grain size than on Mo foil. This suggests that the Mo layer is largely inconsequential to wire formation in contrast to the surface roughness. Wire formation has been shown to be roughness dependent in other materials systems such as ZnO [26,27], but it’s crucial role in CdTe wire formation has never previously been established.

**Figure 2:**
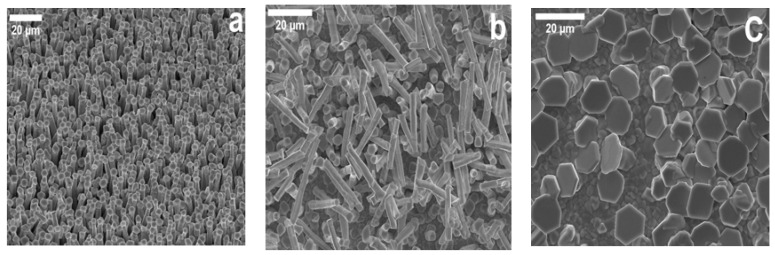
SEM images of CdTe wire growth with varying deposition pressure. (**a**) Vacuum; (**b**) 30 Torr and (**c**) 60 Torr.

In order to determine the level of wire formation control afforded by this self-catalysed route, the influence of CSS deposition pressure was studied as this determines the adatom arrival and re-evaporation rates [28]. Figure 2a–c show SEM images with varying pressure from vacuum (system base pressure i.e., no nitrogen added to ambient) to 30 Torr and to 60 Torr. Deposition was again performed on SLG/Mo to maintain a device relevant stack structure. Layers grown under vacuum exhibit a highly uniform and dense wire array while at 30 Torr the wires become less uniform and are more randomly distributed (11 wires/400 μm^2^ and 5 wires/400 μm^2^ respectively). It has previously been established for CSS growth of thin film CdTe that higher deposition pressures favor the formation of larger grains owing to a reduction in the adatom arrival rate at the surface and thus the formation of larger critical sized nuclei [21]. Here, for growth at 60 Torr, this has led to the formation of excessively large seed crystals, 12.3 ± 0.9 μm, compared to 3.5 ± 0.3 μm for vacuum conditions, which appear unable to effectively template wire growth. There is some evidence of growth occurring beneath these caps, but true wire growth does not occur. These results indicate that while there is a relatively wide pressure range over which growth will occur, the dimensions and the quality of the wires may be adjusted. This follows from previous work where the formation of seed particles and wires is inversely proportional to the growth pressure due to a reduction in the vapor concentration [16,29].

**Figure 3:**
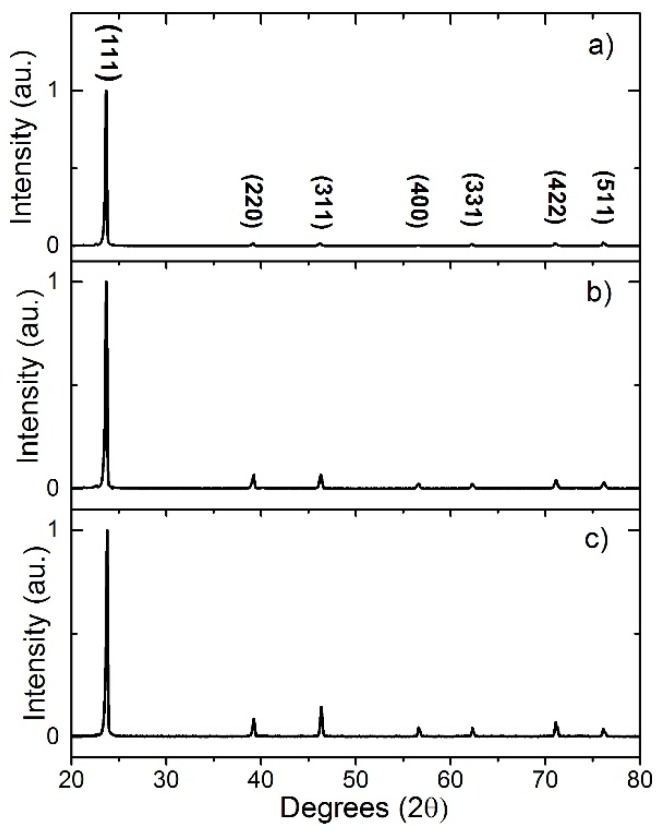
XRD patterns for wire growth on SLG/Mo at (**a**) vacuum and (**b**) 30 Torr; (**c**) Shows XRD pattern of a planar film deposited at 30 Torr on SLG/FTO/Mo.

The XRD patterns shown in Figure 3 were recorded for wires grown on SLG/Mo at either 30 Torr or under vacuum and compared with a planar film deposited on SLG/FTO/Mo. The growth preference may be determined by calculating the texture coefficients (*C*_hkl_) for each diffraction peak using Equation (1).

(1) $$C_{\mathrm{hkl}\;}=\frac{\frac{I_{\mathrm{hkl}}}{I_{r\mathrm{hkl}}}}{1/n_{p}\sum_{n_{p}=1}^{n_{p}}\frac{I_{\mathrm{hkl}}}{I_{r\mathrm{hkl}}}}$$

where *I*_hkl_ is the intensity of a (hkl) diffraction peak, *I_r_*_hkl_ is the relative intensity of this diffraction peak for a powder sample and *n*_p_ is the number of reflections present in the sample. Then a standard deviation (σ) indicates the extent to which the film deviates from the powder using Equation (2) [30]. A high σ indicates the film is more textured and a low value indicates it is more random [30].

(2) $$\sigma \;=\;\sqrt{\sum^{\text{​}}1/n_{p}{\left(C_{\mathrm{hkl}}-1\right)}^{2}}$$

Table 1 shows the *C*_hkl_ and σ for all the samples presented in Figure 3. All samples show a typical diffraction pattern for zinc blende CdTe with a preferential (111) orientation however, the degree of preferential orientation varies. Layers grown on SLG/FTO/Mo (Figure 3c), which have no evidence of wire formation, are the most randomly orientated film σ = 1.59. Both wire samples grown on SLG/Mo show an increase in preferred (111) orientation to σ = 1.83 for growth at 30 Torr (Figure 3b) and σ = 2.23 for growth at vacuum (Figure 3a). This increase would indicate that the wires grow in a (111) orientation.

**Figure 4:**
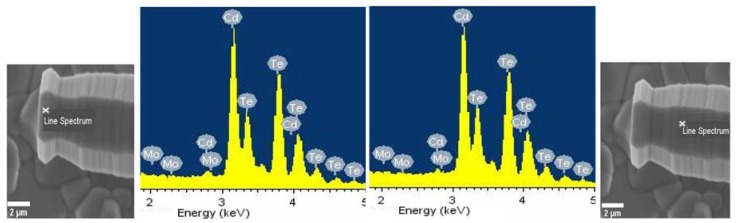
SEM images of the CdTe wires deposited under vacuum on Mo/SLG and EDX spectra at different points of the wires.

One possibility was that the wires were being nucleated by some contaminant on the surface. Although the substrates were thoroughly cleaned prior to deposition, outdiffusion of chemical species from the glass can still occur. To verify that contaminants were not nucleating wire growth, EDX was performed at a number of points along the wires (Figure 4). Spectra were taken from the both the cap and shaft of the wire with the chemical composition being compared. The spectra in both cases looks identical, being predominantly Cd and Te with only small additional peaks from the Mo under layer and O. This indicates that the wires are phase pure CdTe with the additional oxygen being detected always being present in CSS deposited CdTe. The EDX and XRD data presented support the inference that the wires are self-catalysed from a CdTe seed particle which subsequently become the cap of the wire [16].

**Figure 5:**
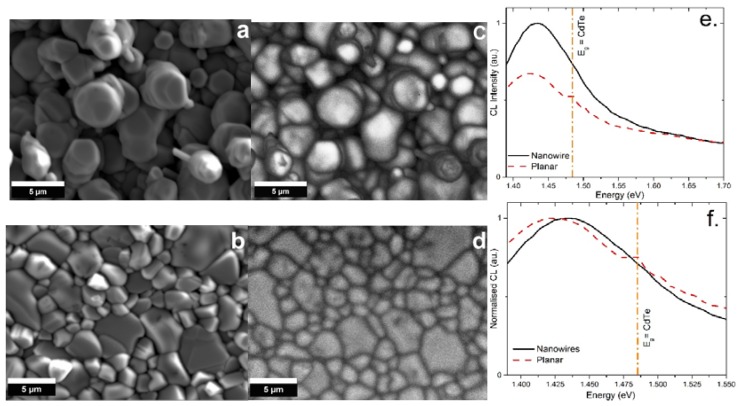
SEM images for CdTe wires (**a**) and the planar (**b**) CdTe film; along with CL images for the wires (**c**) and planar film (**d**). Normalized CL spectra produced for the CdTe wires and CdTe planar film (**e**,**f**). CdTe samples were grown at vacuum. (**e**) Has been normalized with respect to the highest signal, therefore the wire signal was set to 1. In order for the peak shift to be directly analyzed the signals were both normalized to 1 so that the intensities of the signals was neglected (**f**).

It is anticipated that wires should have enhanced carrier transport properties and thus reduced carrier recombination. CL analysis was performed to compare the recombination rates of CdTe wires and thin film. Figure 5a,b show SEM images of the wire and planar layers grown at vacuum on SLG/Mo and SLG/FTO/Mo respectively, with accompanying CL images shown in Figure 5c,d. The CL spectra were collected and normalized in the region of 1.40 to 1.70 eV (Figure 5e). The wires produced a higher signal compared to the planar film indicating the wires possess a reduced level of non-radiative recombination, possibly to a reduction in the number of grain boundaries present in the wires compared to the planar film [31]. This reduced non-radiative recombination makes these ideal for many device applications such as radiation detectors and PV. In order for a direct comparison between the CL signals both were renormalized (Figure 5f) to their respective highest intensity signals, both CL spectra for the wires and planar film show a near band edge transition [11]. The peak for wires is shifted to a slightly higher energy of 1.435 eV compared to 1.419 eV for the planar film, these peaks correspond to a typical donor-acceptor pair transition observed for CdTe [3,5]. The CL spectra for planar films also shows an additional peak at 1.486 eV corresponding to a near band edge transition for CdTe.

## 4. Conclusions

Self-catalysed CdTe wires have been fabricated in a single step technique using a simple industrially scalable PVD technique (CSS). We have produced samples on a SLG/Mo substrate suitable for cell fabrication and demonstrated wire growth dimensions can be controlled by both the surface roughness and deposition pressure. XRD and EDX results indicate that the wires are preferentially (111) oriented and free from contaminants while CL analysis shows reduced recombination in the wires compared to thin films. This development of catalyst free wires on a useable substrate is important for semiconductor applications as it excludes extrinsic growth templating materials which induce deep defects into the CdTe like Bi_Te_ and Au_Cd_ which are detrimental to performance [12]. The next step will be to incorporate these SLG/Mo/CdTe wire structures into complete solar cell devices.

## Figures and Tables

**Table 1:** Texture coefficients (*C*_hkl_) and standard deviation (σ) calculated for each of the samples shown in Figure 3.

	Under Vacuum SLG/Mo	30 Torr SLG/Mo	30 Torr SLG/FTO/Mo
***C*_111_**	6.47	5.511	4.90
***C*_220_**	0.11	0.407	0.43
***C*_311_**	0.10	0.398	0.70
***C*_400_**	0.011	0.144	0.23
***C*_331_**	0.075	0.134	0.21
***C*_422_**	0.11	0.234	0.35
***C*_511_**	0.12	0.171	0.19
**σ**	2.23	1.83	1.59

## Data Availability

The data which supports the findings of this work is available from Liverpool’s Data Catalogue or from the author.

## Supplementary Materials

The following are available online at http://www.mdpi.com/2079-4991/8/5/274/s1, Figure S1 shows the three-dimensional AFM image of the substrates used in this study in order to determine their surface roughness.
