# Supplementary material for: Self-Catalyzed CdTe Wires
Source: Nanomaterials (Basel). 2018 Apr 25;8(5):274. doi: 10.3390/nano8050274 (PMC5977288; doi:10.3390/nano8050274)
Supplement: Supplementary file 1 [file nanomaterials-08-00274-s001.pdf]

Self-Catalyzed CdTe Wires

Tom Baines [1,*], Giorgos Papageorgiou [1,+], Oliver S. Hutter [1], Leon Bowen [2], Ken Durose [1] and Jonathan D. Major [1]

[1] Stephenson Institute for Renewable Energy, Physics Department, University of Liverpool, Liverpool L69 7XF, UK; g.papageorgiou@swansea.ac.uk (G.P.); O.S.Hutter@liverpool.ac.uk (O.S.H.); dph0kd@liverpool.ac.uk (K.D.); jonmajor@liverpool.ac.uk (J.D.M.)

[2] Department of Physics, G.J. Russell Microscopy Facility, Durham University, South Road, Durham DH1 3LE, UK; leon.bowen@durham.ac.uk

* Correspondence: tbaines@liverpool.ac.uk

\+ Now at CTF SOLAR GmbH, Zur Wetterwarte 50, 01109 Dresden, Germany

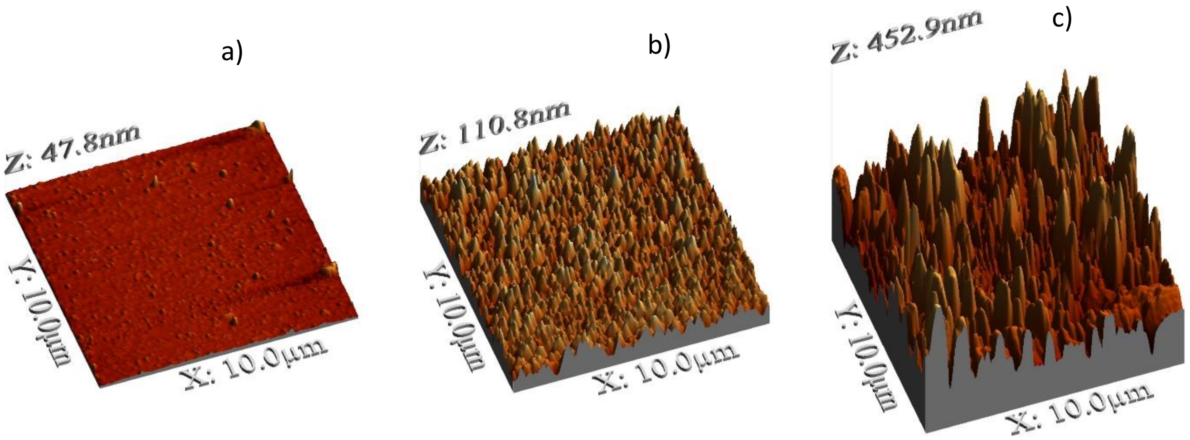

Figure S1: Three dimensional AFM images of the different substrates used, a), SLG/Mo (RMS = 8.0 nm) b), SLG/FTO/Mo (RMS = 16.17 nm) and c), Mo foil (RMS = 71.98 nm).
